# Simultaneous spectrophotometric eco-friendly analysis of triple-drug *H.pylori* regimen (Vonoprazan, Amoxicillin, Metronidazole) for quality control and in vitro dissolution testing

**DOI:** 10.1038/s41598-026-43116-4

**Published:** 2026-04-20

**Authors:** Abdallah M. Hamdy, Radwa I. Abu-Bakr, Soad S. Abd El-Hay, Rania A. Sayed

**Affiliations:** 1 Pharmaceutical Analytical Chemistry Department, Faculty of Pharmacy, Egyptian Russian University, Badr City, Cairo, 11829 Egypt; 2 Pharmaceutical Analytical Chemistry Department, Faculty of Pharmacy, Zagazig University, Zagazig, 44519 Egypt

**Keywords:** Vonoprazan, Amoxicillin, Metronidazole, Dual Wavelength in Ratio Spectra (DWRS), Double Divisor Ratio Spectra Derivative (DDRS-DS), Mean Centering of Double Divisor Ratio Spectra (MC-DDRS), Chemistry, Drug discovery, Gastroenterology, Microbiology

## Abstract

The bacterium *Helicobacter pylori* is considered a major factor in the pathogenesis of gastric ulcers, gastrointestinal disorders, and gastric cancer, leading to the approval of multiple eradication strategies. Among these is the triple therapy regimen comprising vonoprazan, a potassium-competitive acid blocker, with the antimicrobials amoxicillin and metronidazole. This study introduces Dual-wavelength in ratio spectra, Double Divisor Ratio Spectra Derivative, and Mean Centering of Double Divisor Ratio Spectra methods as advanced spectrophotometric techniques that effectively resolved overlapping spectra of the studied mixture. It is the first instance of using these methodologies to determine the cited mixture. The concentration ranges analyzed were 2–60 µg/mL, 2–32 µg/mL, and 2–25 µg/mL for vonoprazan, amoxicillin, and metronidazole, respectively. Greener non-organic solvent was used to ensure environmental sustainability of analytical methods, significantly reducing ecological and health hazards. Greenness assessment was evaluated using GAPI and AGREE metrics, and blueness was assessed by BAGI. One-way ANOVA comparative assessment revealed no statistically significant differences from the reported method. Validation parameters were evaluated according to ICH guidelines, demonstrating sensitivity and selectivity; therefore, the methods are suitable for routine quality control analysis of the analytes in their ternary mixture and pharmaceutical formulation without prior interference from excipients.

## Introduction

*Helicobacter pylori (H. pylori)* is a Gram-negative, microaerophilic bacterium that invades the stomach’s mucosal layer by penetrating the mucus with its flagella and reaching the epithelial cells, surviving by producing urease that neutralizes stomach acid. *H.pylori* remains a major health concern, especially in developing regions like Africa, the Middle East, and South America, where infection rates are still high and a major cause of gastric ulcers, other gastrointestinal diseases, and gastric malignancy^1–3^. The burden of *H.pylori* infection has progressively lessened globally, evidenced by a reduction in prevalence from 52.6% before 1990 to 43.9% in contemporary adult populations (2015–2022)^4^. This decrease is attributed to improved sanitation, living conditions, and better treatment options. However, infection rates among children and adolescents have remained largely unchanged despite the decline^5^. Regimens using Vonoprazan and antimicrobial combination therapy^6,7^ are an effective and promising therapy for *H.pylori* eradication treatment^8,9^. The combination of Vonoprazan, Amoxicillin, and Metronidazole has been approved as the optimal option for secondary infections^10^ and patients showing resistance to Clarithromycin^11^.

**Fig. 1:**
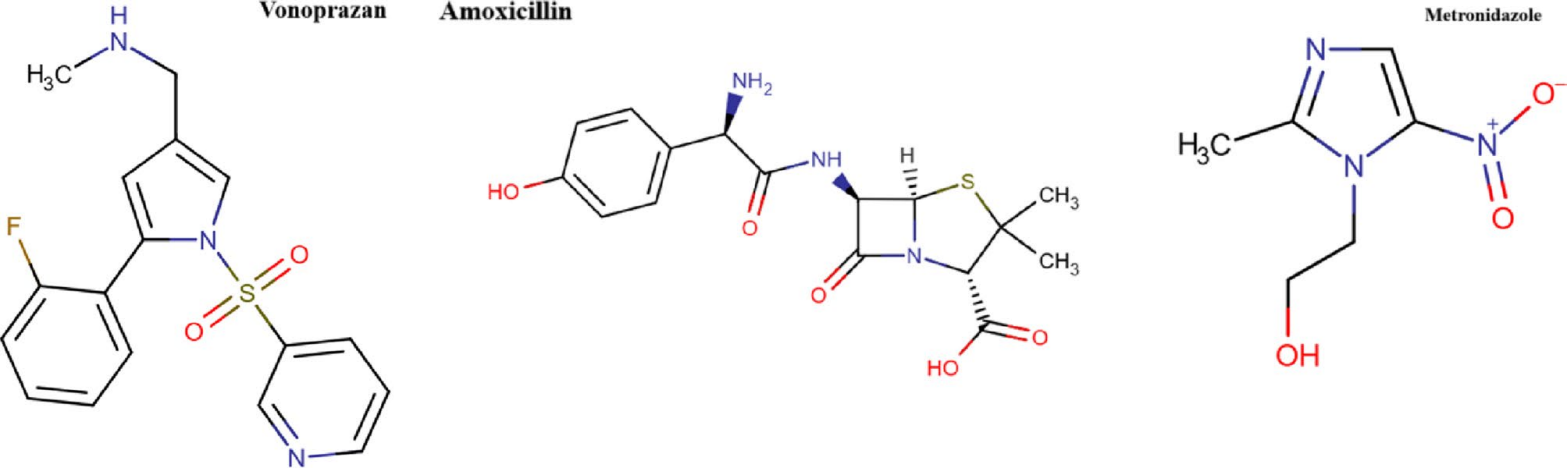
Structures of Vonoprazan, Metronidazole and Amoxicillin.

Vonoprazan (VON) Fig. 1 functions as Potassium-Competitive Acid Blockers (PCABs) that inhibits the H⁺/K⁺-ATPase proton pump reversibly, the enzyme in charge of gastric acid secretion. Unlike proton pump inhibitors(PPIs), PCABs serve as an alternative treatment for acid-related disorders by reversibly blocking acid production^8,12^. PCABs is that CYP2C19 genetic polymorphisms, which can alter PPI metabolism, have no effect on their effectiveness^13^. Furthermore, PCABs are stable in the stomach’s acidic environment, so they don’t need formulations resistant to acid, guaranteeing consistent absorption and efficacy^14^.

Amoxicillin (AMX), Fig. 1, is a broad-spectrum β-lactam antibiotic in the penicillin class, derived semi-synthetically from ampicillin. It has improved oral bioavailability and a broader antibacterial spectrum. It is indicated for bacterial infections of the ENT (ear-nose-throat) regions, genitourinary system, dermal surfaces, and lower respiratory system^15^. Additionally, it is used with omeprazole or vonoprazan as a co-packaged dual therapy for H. pylori treatment.

Metronidazole (MET), Fig. 1, is a nitroimidazole antibiotic with a 5-nitroimidazole ring crucial for its antimicrobial efficacy. It exhibits high effectiveness against anaerobic bacteria and protozoa^16,17^. As a prodrug, metronidazole becomes activated under anaerobic conditions. Within bacterial or protozoal cells, it undergoes reduction by ferredoxin or nitroreductase enzymes, forming reactive nitrogen species^18^.

The literature presents a range of UV spectrophotometric analytical methods for analyzing VON either separately^19^, For VON combined with other drugs such as AMX^20^, Methods for analysis of AMX separately^21,22^ or combined with other drugs such as MET^23^, Methods for determination of MET^24^.

HPLC method has been designed as an approach for simultaneously determining this ternary mixture, while no spectrophotometric methods have been proposed for resolving this mixture^25^.

This study aims to develop eco-friendly UV spectrophotometric techniques that enable accurate and efficient simultaneous quantification of Vonoprazan, Amoxicillin, and Metronidazole. This method is highly beneficial for ensuring drug quality in pharmaceutical manufacturing and assists in analyzing dissolution behavior for pharmacokinetic studies. A further application is the dissolution test as an essential quality control assay that serves as a vital tool in verifying batch uniformity, predicting in vivo bioavailability, and ensuring the therapeutic efficacy of the drug product.

## Experimental

### Apparatus and software

Jasco (Japan) V-630 UV/VIS spectrophotometer was employed for spectroscopic measurements, and the spectral data were obtained using Jasco Spectra Manager. Spectrophotometric measurements were performed between 200 nm and 400 nm to characterize the absorption spectra of the sample and reference solutions. Elma Schmidbauer® Elmasonic Easy 100 H sonicator. pH meter model Jenway® 4510 scientific, UK. Copley® Scientific DIS 6000 is equipped with six stirred test vessels, organized in two rows of three, enabling effective dissolution testing of solid dosage forms. The regression equations were developed using Microsoft Excel®, while the mean data-centering calculations were performed using the MATLAB® program.

### Materials and solvents

High-purity standards were sourced as Vonoprazan fumarate (VON): 99.6% (P&C Laboratory, 10th of Ramadan City, Egypt), Amoxicillin trihydrate (AMX): 99.8% and Metronidazole (MET): 99.8% (Eipico, 10th of Ramadan City, Egypt) all standards are supplied from National Organization for Drug Control and Research. Vonopion® (labeled to contain 20 mg VON, 250 mg AMX, and 250 mg MET) was manufactured by Takeda Pharmaceutical Company Limited (Tokyo 103–8668, Japan).

Simulated gastric fluid prepared (SGF) by dissolving 3.0 gm sodium chloride in 1500 ml of distilled water and pH is adjusted to1.2 ± 0.1, titrated by 0.1 N hydrochloric acid (HCl) using NaCl (99.9%) and HCl (32%) assayed according to the manufacturer (Piochem for laboratory chemicals, 1st industrial zone, Street no.3, 6th of October city, Giza, Egypt.)^26^.

### Standard solutions preparation

Standard solutions were prepared with the following concentrations: 100 µg/mL VON, 200 µg/mL AMX, and 100 µg/mL MET. The stock solutions were prepared in three separate 100 mL volumetric flasks by adding 10 mg of VON, 10 mg of MET, and 20 mg of AMX to each flask. The volume was then brought up to the mark with the SGF as solvent.

### Spectral profile of VON, AMX, and MET

**Fig. 2:**
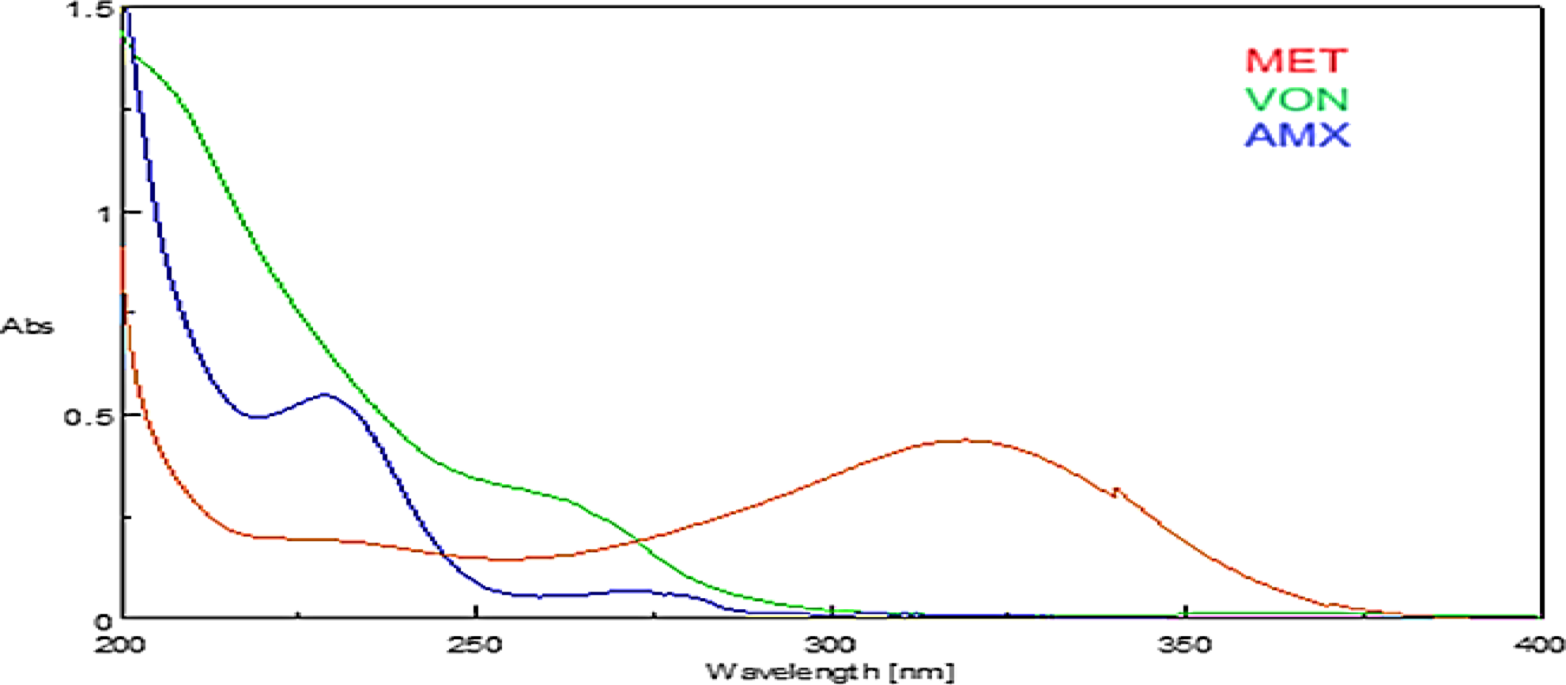
Overlaying UV spectra of, (10 µg/mL) VON, (10 µg/mL) AMX and (5 µg/mL) MET against SGF as blank.

Aliquots from the stock solutions were accurately transferred into three 10 mL volumetric flasks. Each flask was filled to the mark with SGF. The zero-order (D_0_) absorption spectrum was subsequently recorded, using SGF as a blank, as shown in Fig. 2.

### Dual wavelength in ratio spectra (DWRS) method

To determine VON, pipetting aliquots of the 100 µg/mL stock standard solution into a series of 10 mL volumetric flasks was done one at a time. SGF was then added to each flask until it reached the mark corresponding to a range (2–60) µg/mL of VON, The solutions were analyzed, 2 µg/mL MET chosen as a divisor, and the amplitude difference at 260.8 and 272.8 nm in the ratio spectrum obtained using (${\varDelta\mathrm{A}}_{260.8-272.8\mathrm{n}\mathrm{m}}^{\mathrm{M}\mathrm{E}\mathrm{T}2{\upmu}\mathrm{g}/\mathrm{m}\mathrm{L}}$) was plotted versus the corresponding concentrations of VON to construct the calibration graph, and a regression equation was obtained^27^.

For AMX, Serial dilutions from 200 µg/mL stock were prepared similarly to obtain concentrations in the range of (2–32) µg/mL. Spectra were divided by a divisor 2 µg/mL of MET. The ratio spectrum’s amplitude difference at wavelengths of 210.2 and 226 nm was determined using ($\varDelta{A}_{210.2-226nm}^{MET2{\upmu}g/mL}$). The ratio spectra were correlated with AMX concentrations, establishing calibration graphs and obtaining a regression equation.

For MET, a series of standard solutions (2–25) µg/mL was prepared by dilution from a 100 µg/mL stock standard solution, adhering to the prescribed analytical protocol. These solutions’ absorption spectra were divided by 14 µg/mL of VON spectral reference. Differential absorbance (ΔA) was measured at 279.8 nm and 303 nm selected based on the isosbestic behavior of AMX. A linear calibration model was subsequently derived by regressing ($\varDelta{A}_{279.8-303nm}^{VON14\mu g/mL}$) against MET concentrations, and a regression equation was obtained.

### Double Divisor Ratio Spectra Derivative Spectrophotometric [DDRS-DS] method

For VON, the absorption spectra within the range of (2–60) µg/mL were divided by the combined spectra of AMX (14 µg/mL) and MET (14 µg/mL), acting as a “double divisor,” thereby obtaining the ratio spectra. Subsequently, the first derivative of these ratio spectra was obtained. The amplitudes measured at the maximum wavelength of 223 nm were exclusively dependent on concentration values of VON, so calibration curves and corresponding regression equations were calculated.

For AMX, absorption spectra of standard solutions (2–32) µg/mL were processed using a double-divisor approach, wherein spectra were divided by the combined spectra of VON 14 µg/mL and MET (14 µg/mL). First-derivative conversion was then applied to the obtained ratio spectra and the derivative amplitudes at 240.4 nm were measured in order to determine the AMX concentrations, where the signal showed dependence on AMX concentrations, and calibration curves and corresponding regression equations were obtained.

For MET, the absorption spectra within the range of (2–25) µg/mL were divided by the combined spectra of VON (26 µg/mL) and AMX (26 µg/mL), serving as a “double divisor,” thereby generating the ratio spectra. Subsequently, the first derivative of these ratio spectra was calculated. The amplitudes measured at the maximum wavelength of 339.8 nm were exclusively dependent on the concentration values of MET.

### Mean centering of Double Divisor Ratio Spectra Spectrophotometric [MC-DDRS] method

In the analysis of VON, absorption spectra within the concentration range of (2–60) µg/mL were normalized using the combined spectra of AMX and MET, each at a concentration of 14 µg/mL, as a ‘double divisor’, thereby obtaining the ratio spectra. These ratio spectra were subsequently mean-centered utilizing MATLAB; the observed amplitudes measured at the maximum wavelength of 215.6 nm were exclusively dependent on the concentration values of VON.

The same procedures were followed for AMX, absorption spectra within the concentration range of (2–32) µg/mL were normalized using the combined spectra of VON and MET, each at a concentration of 14 µg/mL, as a ‘double divisor’. The maximum wavelength was 231.2 nm.

For MET, absorption spectra within the concentration range of (2–25) µg/mL were normalized using the combined spectra of VON and AMX, each at 26 µg/mL, as a ‘double divisor’, and the maximum wavelength is 322.6 nm.

The calibration curves of the three drugs were established by plotting the amplitude value at the maximum wavelength of their respective mean-centered ratio spectra against their corresponding concentrations.

### Analysis of laboratory-prepared mixtures

Three sets of prepared mixtures were analyzed at the following ratios: 10:10:10, 16:24:8, and 2:8:2, respectively, and were prepared and analyzed according to the procedures outlined for each proposed method. Each drug concentration was determined from the computed regression equation of each technique. Additionally, laboratory-prepared mixtures were developed in ratios corresponding to those found in the marketed formulation.

### Standard addition technique

This method was developed as an innovative enrichment strategy to address challenges in analyzing trace components in mixtures^28–30^. The standard addition method is an analytical technique used to quantify the concentration of an analyte in a complex sample, where interference from other components may affect accuracy. It involves adding known increments of a standard solution to the sample and measuring the response. This method enables precise quantification of target analytes in complex matrices where interfering components may affect direct measurements.

The technique involves sequential addition of known analyte concentrations of standard to the sample, followed by measurement of the corresponding analytical response at each increment^31^.

### Analysis of pharmaceutical preparations

Ten dose forms of VONOPION® a triple-medication blister pack intended for second-line treatment labelled 20 mg Vonoprazan fumarate, 250 mg Amoxicillin hydrate, and 250 mg Metronidazole. The average weight of 10 dosage forms was calculated, and then they were crushed and thoroughly blended in a mortar to guarantee good homogeneity. The average weight of one dosage form of each VON, AMX, and MET was transferred into a 100 mL volumetric flask, and a volume of 50 mL SGF was added. The resulting mixture was sonicated for 15 min, followed by volume adjustment to mark 100 ml, then filtration using a 0.45 μm sterile syringe filter disc. 0.1 mL was pipetted into volumetric flask 10 mL, and volume was adjusted with SGF to the mark.

### Dissolution of pharmaceutical preparations

The dissolution test demonstrates the ability of the developed analytical methods to simultaneously quantify the three drugs within a complex and in a clinically relevant medium, also plays a crucial role in validating the bioavailability and therapeutic performance of oral formulations. Accordingly, it confirms that all three active components are released from their respective tablets or capsules in a timely and complete manner, supporting their effectiveness for treatment of H. pylori infection.

The dissolution study used a Copley Scientific DIS 6000 apparatus. A single tablet of VON (20 mg) and MET (250 mg), along with three capsules of AMX (250 mg), were introduced into 1000 mL of simulated gastric fluid pH 1.2 as the dissolution medium. The apparatus was set to a rotational speed of 5 RPM to simulate gentle agitation. The temperature was precisely controlled at 37 ± 0.5 °C to mimic physiological conditions. At predetermined intervals of every 30 min till 180 min, 5 mL aliquots were withdrawn, with immediate medium replacement to maintain sink conditions. Sampling continued until complete or near-complete drug release was observed.

## Results and discussion

Vonoprazan exhibits a UV absorption spectrum that significantly overlaps with that of Amoxicillin and Metronidazole; this spectral overlap complicates direct spectrophotometric quantification, Because there is no distinct wavelength region or zero-crossing point that enables the selective detection of any component without significant interference from the other two, conventional UV spectrophotometry cannot be employed effectively, Additionally, higher-order derivatives led to severe noise amplification and sensitivity loss, which resulted in low accuracy and repeatability, making derivative spectrophotometric methods inappropriate for this ternary system.

Thereby necessitating the use of advanced spectrophotometric and mathematical techniques for resolution, as depicted in Fig. 2. Consequently, direct spectrophotometric methods are inadequate for accurately identifying individual components within their mixture. Conversely, the newly developed spectrophotometric techniques have shown proficiency in resolving the overlapping spectral data of these three constituents. Validation through practical application on ternary mixtures confirmed the ability of these methods to determine each component selectively without interference from the others.

### Methodology development and optimization

The three proposed methods were developed for determining ternary mixtures. Since method development is a detailed process where testing methods are carefully designed and checked to meet necessary performance standards, such as how well they can detect substances, resist interference, measure accurately, and work reliably, ensuring they are suitable for their intended use.

### Dual wavelength in ratio spectra (DWRS) method

The principle behind this method is to eliminate the interference caused by the two components while identifying one analyte in a ternary mixture^32^. A horizontal straight-line ratio spectrum with zero difference between two wavelengths is obtained using Component A as a divisor to eliminates its effect the interference between B and C is eliminated by choosing two wavelengths at which component B has precisely the same amplitude in the same ratio spectrum so Absorbance difference (A(λ1) - A(λ2)) can be used quantitatively for C^33^.

The suggested approach starts by scanning the zero-order spectra of the VON, MET, and AMX standard solutions. Afterward, various divisor concentrations were tested. Carefully choosing the divisors will allow you to balance optimum sensitivity and low noise. Different wavelengths on the ratio spectra were also selected and tried. Choosing the divisors and wavelengths carefully balances low noise and optimum sensitivity.

**Fig. 3:**
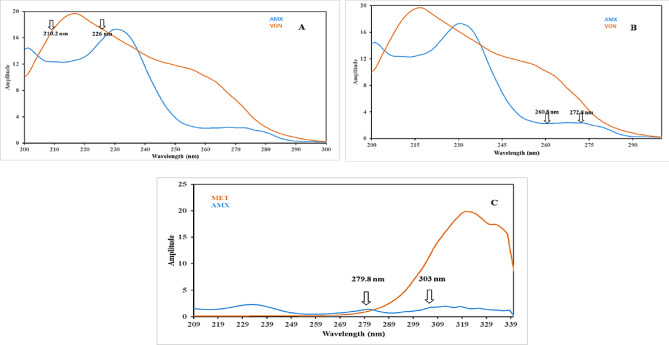
*(***A)** is Ratio spectra of VON (14 µg/mL), AMX (20 µg/mL) divided by MET (2 µg/ml) shawing wavelengthes to detect Vonoprazan, **(B)** is Ratio spectra of VON (14 µg/mL), AMX (20 µg/mL) divided by MET (2 µg/ml) shawing wavelengthes to detect Amoxicillin, (**C)** is Ratio spectra of AMX (26 µg/mL), MET (2 µg/mL) divided by VON (14 µg/mL) shawing wavelengthes to detect MET.

The optimum divisor concentrations were (2 µg/mL) MET for VON and AMX prediction. While for MET, 14 µg/mL VON divisor was the divisor of choice. Regarding the selected wavelengths, the wavelengths (260.8 and 272.8 nm) were used for VON determination, at which AMX is consistent, as shown in Fig. 3A. For AMX, 210.2 nm and 226 nm were selected as VON-invariant wavelengths, as shown in Fig. 3B. While for MET, the chosen wavelengths were 279.8 and 303 nm, where AMX exhibits the same amplitude, as shown in Fig. 3C. The peak amplitude differences at the selected wavelengths for each drug were recorded and correlated with each concentration.

The regression equations were calculated as follows:

1 $${\varDelta\mathrm{A}}_{260.8-272.8\mathrm{n}\mathrm{m}}^{\mathrm{M}\mathrm{E}\mathrm{T}2{\upmu}\mathrm{g}/\mathrm{m}\mathrm{L}}= 0.3349\; C_{VON} + 0.1849\; for\; VON$$

2 $$\varDelta{A}_{210.2-226nm}^{MET2\mu g/mL}= 0.1820\; C_{AMX} + 0.2235 \;for\; AMX$$

3 $$\varDelta{A}_{279.8-303nm}^{VON14\mu g/mL}= 3.9511\; C_{MET} + 0.2668\; for\; MET$$

The proposed method was successfully applied to simultaneously determine VON, AMX and MET in their mixtures. The ternary mixture was scanned and then divided by the suitable divisor to obtain the ratio spectra. Afterwards, the amplitude differences were calculated at the selected wavelengths and used to get the drug concentrations from their respective regression equations.

### Double Divisor Ratio Spectra Derivative Spectrophotometric [DDRS-DS] method

This methodology utilizes ratio spectrum transformation, where the absorption spectrum of the ternary mixture is divided by a binary reference spectrum (containing two components). This division can effectively cancel their spectral interference and enable selective analysis of the remaining third component^34^. Then, applying the first derivative to the ratio spectrum can remove background interference and overlapping spectral contributions. The concentrations of the three compounds in the mixture are then determined using their respective calibration graphs, created by measuring the amplitude at either the maximum or minimum selected wavelengths^35–37^.

**Fig. 4:**
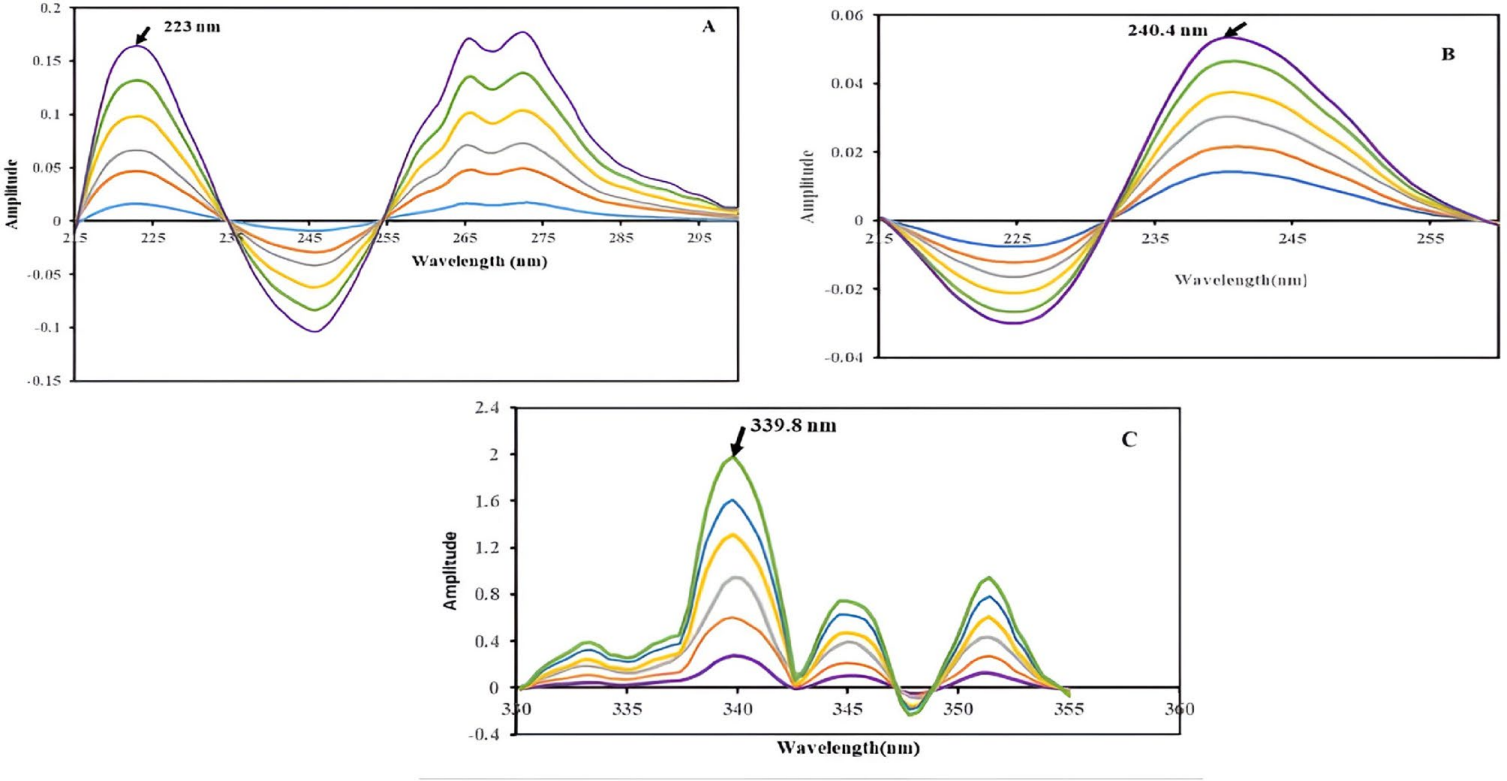
**(A)** is First derivative of the ratio spectra of VON (2–60 µg/mL) using 14 µg/mL of each of AMX and MET as a double divisor, (**B)** is First derivative of the ratio spectra of AMX (2–32) µg/mL) using 14 µg/mL of each of VON and MET as a double divisor, (**C)** is First derivative of the ratio spectra of MET (2–25) µg/mL using 26 µg/mL of each of VON and AMX as a double divisor.

Since the choice of divisors and their concentrations is essential, several concentrations of VON, AMX, and MET were investigated as divisors. Divisors and binary mixtures of AMX/MET, VON/MET, and VON/AMX at equal and different concentrations were investigated. The best results were obtained in terms of selectivity, sensitivity, and signal-to-noise ratio. by using a mixture of equal concentrations of AMX/MET, VON/MET (both 14 µg/mL of each), and VON/AMX (26 µg/mL of each) as divisors for determining VON, AMX, and MET, respectively. So, ratio spectra were generated, followed by derivatization. The first derivative of these ratio spectra yielded concentration-dependent amplitudes at 223, 240.4, and 339.8 nm for VON, AMX, and MET, respectively, Fig. 4A and B, and C.

The calibration data yielded the following regression equations:

4 $${\mathrm{A}}_{{{\mathrm{VON}}}} = {\text{ }}0.00{\text{27 C}}_{{{\mathrm{VON}}}} - {\text{ }}0.00{\text{27 at 223nm for VON}}$$

5 $${\mathrm{A}}_{{{\mathrm{AMX}}}} = {\text{ }}0.00{\mathrm{2}}0{\text{ C}}_{{{\mathrm{AMX}}}} + {\text{ }}0.00{\text{21 at 24}}0.{\text{4nm for AMX}}$$

6 $${\mathrm{A}}_{{{\mathrm{MET}}}} = {\text{ }}0.{\text{1134 C}}_{{{\mathrm{MET}}}} + {\text{ }}0.0{\text{442 at 339}}.{\text{8nm for MET}}$$

The suggested approach effectively determined their mixtures’ VON, AMX, and MET simultaneously. The ratio spectra were obtained by scanning the ternary mixture and then dividing it by the appropriate double divisor, followed by derivatization. The drug concentrations were then obtained from their corresponding regression equations using the peak amplitudes at the chosen wavelengths.

### Mean centering of Double Divisor Ratio Spectra Spectrophotometric [MC-DDRS] method

**Fig. 5:**
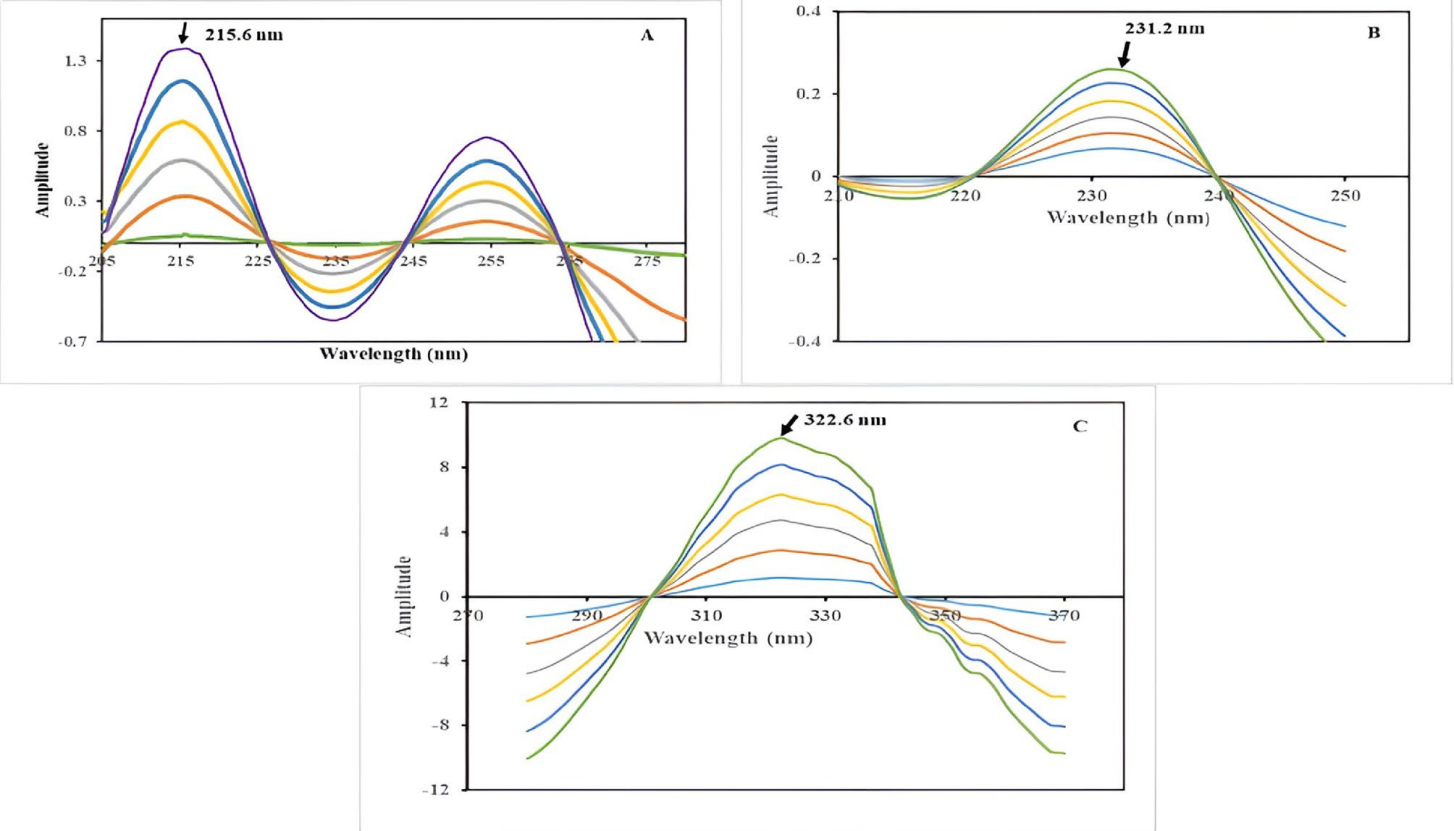
**(A)** is Mean centering technique showing amplitudes of VON (2–60) µg/ml using 14 µg/mL of both MET and AMX as a divisor at maximum wavelength 215.6 nm, (**B)** is Mean centering technique showing amplitude of AMX (2–32) µg/mL l using 14 µg/mL of both MET and VON as a divisor at maximum wavelength 231.2 nm, C is Mean centering technique showing amplitude of MET (2–25) ug/ml using 26ug/mL of both AMX and VON as a divisor at maximum wavelength 322.6 nm.

This approach uses mathematical transformations to enhance spectral resolution and quantification accuracy. It combines the Double Divisor Ratio Spectra (DDRS) method with Mean Centering (MC) by dividing the absorption spectra of the ternary mixture by the sum of two standard spectra a double divisor, the obtained ratio spectra are processed and Calibration curves were constructed to establish the relationship between the mean-centered data, obtained using the MATLAB^®^ 7.0.1 Software program function at the selected wavelengths, and the corresponding concentrations^38,39^. The VON, AMX, and MET concentrations were determined by measuring the amplitudes at 216, 231.6, and 322.6, respectively, as shown in Fig. 5A and B, and C. Subsequently, regression was derived to quantify these equations:

7 $${\mathrm{A}}_{{{\mathrm{VON}}}} = {\text{ }}0.0{\text{219 C}}_{{{\text{VON }} + }} 0.0{\mathrm{227}}~~{\text{at }}\lambda {\text{max 215}}.{\text{6 nm for VON}}$$

8 $${\mathrm{A}}_{{{\mathrm{AMX}}}} = {\text{ }}0.00{\text{98 C}}_{{{\mathrm{AMX}}}} + {\text{ }}0.00{\mathrm{84}}~{\text{at }}\lambda {\text{max 231}}.{\text{6 nm for AMX}}$$

9 $${\mathrm{A}}_{{{\mathrm{MET}}}} = {\text{ }}0.{\text{5789 C}}_{{{\mathrm{MET}}}} + {\text{ }}0.0{\mathrm{192}}~~{\text{at }}\lambda {\text{max 322}}.{\text{6 nm for MET}}$$

### Method validation parameters

The International Council for Harmonization (ICH) specifies key criteria that must be assessed when validating analytical methods to confirm their reliability and appropriateness for their intended use. The validation process for these methods encompasses testing for linearity, accuracy, and precision, as well as determining detection limits (LOD), quantification limits (LOQ), and selectivity^40^.

### Linearity

VON, AMX, and MET calibration curves were constructed. Each standard solution was analyzed in triplicate across all proposed methods, plotting responses (absorbance or amplitude) against concentration to generate optimal linear regression lines. Linearity was assessed using the coefficient of linearity, as presented in Table 1. Values demonstrated that the developed methods were suitable for quantifying the target drugs across a wide concentration range.

**Table 1:** Method validation characteristics for VON, AMX, and MET according to ICH guidelines.

Method	DWRS			DDRS-DS			MC-DDRS		
Parameter	VON	AMX	MET	VON	AMX	MET	VON	AMX	MET
Wavelength	${\varDelta\mathrm{A}}_{260.8-272.8\mathrm{n}\mathrm{m}}^{\mathrm{M}\mathrm{E}\mathrm{T}2{\upmu}\mathrm{g}/\mathrm{m}\mathrm{L}}$	$\varDelta{A}_{210.2-226nm}^{MET2{\upmu}g/mL}$	$\varDelta{A}_{279.8-303nm}^{VON14\mu g/mL}$	223 nm	240.4 nm	339.8 nm	215.6 nm	231.2 nm	322.6 nm
Range	2–60 µg/mL	2–32 µg/mL	2–25 µg/mL	2–60 µg/mL	2–32 µg/mL	2–25 µg/mL	2–60 µg/mL	2–32 µg/mL	2–25 µg/mL
Slope	0.3349	0.1820	3.9511	0.0027	0.0020	0.1134	0.0219	0.0098	0.5789
Intercept	0.1849	0.2235	0.2668	-0.0027	0.0021	0.0442	0.0227	0.0084	0.0192
Correlation coefficient(r)	0.9999	0.9999	0.9999	0.9999	0.9995	0.9997	0.9999	0.9994	0.9998
LOD	0.4869	0.4805	0.1441	0.2007	0.2900	0.0345	0.2414	0.1858	0.2308
LOQ	1.4755	1.4560	0.4367	0.6082	0.8789	0.1046	0.7315	0.5632	0.6996
Accuracy ^a^	99.03	99.48	99.86	101.29	101.22	99.94	101.12	100.77	99.32
Robustness ^b^	0.45	0.72	0.64	0.68	1.33	0.91	1.52	0.41	0.34
Specificity ^c^	99.41 ± 0.57	99.62 ± 1.31	98.51 ± 0.47	100.56 ± 1.42	101.54 ± 0.30	100.53 ± 0.62	99.81 ± 1.08	101.04 ± 0.69	100.45 ± 0.99
Repeatability ^d^	0.78	0.70	0.64	1.70	1.08	0.57	1.40	1.60	0.19
Intermediate precision ^e^	0.86	1.22	0.82	1.34	0.98	1.11	1.77	1.57	0.75

^**a**^ Accuracy was evaluated by using nine measurements at three different concentration levels within the specified range.

^**b**^ Robustness was expressed by RSD of the results obtained by the examination of the effect of three different pH (1.1, 1.2, 1,3) on laboratory prepared mixture.

^**c**^ Specificity was expressed by mean of percentage recoveries ± SD of the laboratory prepared mixtures.

^**d**^ Repeatability was expressed by RSD of three distinct concentrations repeated three times on the same day. The concentrations were (14 µg/mL -26 µg/mL 38 µg/mL) for VON, (8 µg/mL -14 µg/mL -20 µg/mL) for AMX, and (7 µg/mL -17 µg/mL -22 µg/mL) for MET.

^**e**^ Intermediate precision was expressed by RSD of the same three concentrations by repeating the analyses of solutions three times on three different days.

### LOD and LOQ

According to the ICH Q2(R2) criteria, the lower range limits, such as the limit of detection (LOD) and limit of quantification (LOQ), were determined in order to assess the sensitivity of the proposed procedures. The lowest detectable and quantifiable concentrations are presented in Table 1. These values were calculated using the following equations:

10 $$LOD=\frac{3.3\mathrm{*}{\upsigma}}{\mathrm{S}}$$

11 $$LOQ=\frac{10\mathrm{*}{\upsigma}}{\mathrm{S}}$$

Where σ represents the standard deviation of the blank and S represents the slope of the calibration graph. Also, accuracy and precision measurements of the lower range limits were examined.

### Accuracy

Accuracy is determined by comparing measured results with reference values. Evaluation is performed under standard testing conditions, including the presence of sample matrix components. Testing across a range of concentrations within the validated range of the method ensures reliability. Three distinct concentrations are usually analyzed; each is tested in triplicate, resulting in nine total determinations. The results showed that the methods are accurate.

#### Precision

Precision assessment was performed using three concentrations of each drug in triplicate. Intra-day precision (repeatability) was assessed on the same day, and inter-day precision (intermediate precision) was evaluated in three consecutive days. Relative standard deviation (% RSD) values did not exceed 2%, indicating the proposed method’s good precision, as shown in Table 1.

### Robustness

Robustness is ability of a method to tolerate minor variations in analytical parameters. To guarantee that, despite little changes in the experimental conditions, the UV spectroscopic methods provide reliable and accurate results.

Robustness was assessed by evaluating the results obtained by the examination of the effect of change in pH 1.2 ± 0.1. The methods showed good Robustness. The data was representd in Table 1.

### Specificity

Method specificity was verified by analyzing laboratory-prepared mixtures containing varying VON, AMX, and MET ratios. The calculated percentage recoveries (% R) and standard deviation (SD) values demonstrated acceptable specificity of the methods for targeted analytes across all tested compositions, as shown in Tables 1 and 2.

**Table 2:** Quantitative VON AMX and MET analysis in laboratory-prepared mixtures utilizing the proposed methodologies.

Method	DWRS			DDRS-DS			MC-DDR		
VON AMX MET Conc ( µg/mL)	VON *R*%	AMX *R*%	MET *R*%	VON *R*%	AMX *R*%	MET *R*%	VON *R*%	AMX *R*%	MET *R*%
2 8 2	98.57	99.66	98.21	99.22	101.32	100.63	99.36	102.01	100.39
10 10 10	99.65	97.97	98.17	101.97	101.43	100.26	99.11	100.36	99.96
16 24 8	99.60	101.17	99.18	101.21	101.99	101.34	99.34	100.95	101.86
2 25 25	99.82	99.66	98.49	99.85	101.44	99.89	101.41	100.85	99.59
Mean	99.41	99.62	98.51	100.56	101.54	100.53	99.81	101.04	100.45
SD	0.57	1.31	0.47	1.42	0.30	0.62	1.08	0.69	0.99

### Pharmaceutical preparation determination, and application of the standard addition technique

The three proposed spectrophotometric methods were effective for the determination of VONOPION® tablets, showing no excipient interference. Their reliability was verified through standard addition experiments, as the standard addition method is a matrix-matched calibration technique designed to accurately determine analyte concentrations in samples, as shown in Table 3 And quantitative analysis of VON, AMX, and MET in pharmaceutical dosage form is represented in Table 4.

**Table 3:** The application of the standard addition technique for determination of VON, AMX, and METusing the proposed spectrophotometric methods.

Method		VON								AMX										MET			
		DWRS		DDRS-DS		MC-DDR				DWRS		DDRS-DS		MC-DDR				DWRS		DDRS-DS		MC-DDR	
Conc ( µg/mL)		*R*%		*R*%		*R*%		Conc ( µg/mL)		*R*%		*R*%		*R*%		Conc ( µg/mL)		*R*%		*R*%		*R*%	
APP ^a^	added ^b^	APP	added	APP	added	APP	added	APP	added	APP	added	APP	added	APP	added	APP	added	APP	added	APP	added	APP	added
	4		99.71		101.06		99.80		4		100.68		101.73		101.29		3		98.97		99.61		99.57
10	8	99.77	99.03	100.37	102.32	98.89	101.73	10	10	97.93	99.51	99.97	102.56	97.59	99.54	10	5	99.40	99.93	100.23	99.65	97.95	98.52
	12		98.56		100.50		101.81		14		99.45		99.38		101.48		7		100.70		100.56		99.86
Mean		99.03		101.29		101.12				99.48		101.22		100.77				99.86		99.94		99.32	
SD		0.58		0.93		1.14				0.04		1.65		1.07				0.87		0.53		0.71	
RSD		0.58		0.92		1.12				0.04		1.63		1.06				0.87		0.53		0.71	

^a^ Pharmaceutical dosage form.

^b^ Added concentration.

**Table 4:** Quantitative analysis of VON, AMX, and MET in pharmaceutical dosage form by directly applying the proposed spectrophotometric methods.

Method	VON			Conc (μg/mL)	AMX			Conc (μg/mL)	MET		
Conc (μg/mL)	DWRS	DDRS-DS	MC-DDR		DWRS	DDRS-DS	MC-DDR		DWRS	DDRS-DS	MC-DDR
	101.13	100.59	100.71		98.84	98.72	100.36		98.89	99.3	98.6
2	100.51	100.77	101.17	25	99.99	100.93	100.4	25	99.99	99.65	99.01
	100.21	101.15	100.49		100.07	100.13	100.69		101.03	101.06	99.08
Mean	100.61	100.83	100.79		99.64	99.92	100.48		99.97	100.01	98.9
SD	0.47	0.28	0.35		0.69	1.12	0.18		1.07	0.93	0.26

### Dissolution of pharmaceutical dosage forms

**Fig. 6:**
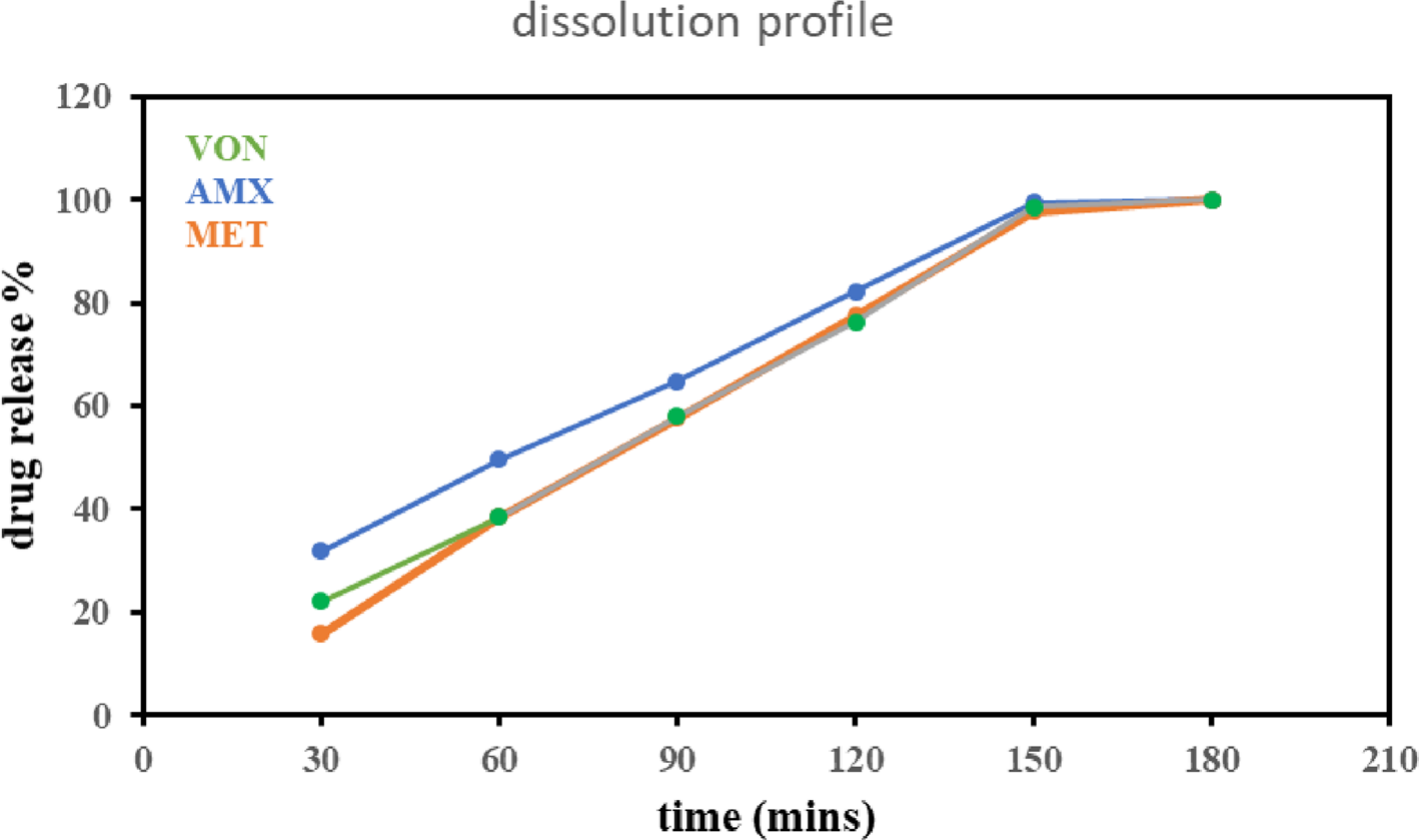
Dissolution profile of Vonopion^®^ contain (20 mg of VON ,750 mg of AMX and 250 mg of MET).

The drug release profile Fig. 6 of VONOPION^®^ shows efficient dissolution in simulated gastric fluid, with all three drugs releasing over 97% within 150 min. This confirms its suitability for ensuring simultaneous drug availability and stability under gastric conditions, a key factor for effective absorption and therapeutic response.

The stability behavior of VON and MET in simulated gastric fluid (SGF) has been well documented in previous research, in accordance with these findings, both stock and working standard solutions of VON and MET were prepared using SGF as the diluent, as previous studies confirmed that these compounds remain chemically stable under acidic conditions and can be stored under refrigeration for several days without significant degradation. AMX) stock and working solutions prepared in simulated gastric fluid (pH 1.2) have been reported to retain more than 90% of their initial concentration for approximately 6–8 h. The stability was specifically verified under the experimental conditions employed in this study for and proved good stability with time regarding relative standard deviation^41–44^.

In the VONOPION^®^ formulation, the medicinal dose ratio was (2:75:25) of (VON: AMX: MET), respectively. To detect VON as a minor component simultaneously with the other two components, considering the linearity range of each drug, so, applying the enrichment technique was necessary. Enrichment of VON was performed by adding a known concentration of VON (10 µg/mL). The VON concentration in the dosage form is obtained by subtracting the added VON amount from the sample’s total drug concentration.

### Statistical comparison of the proposed methods with the reported method

Statistical comparison using one-way ANOVA at a 95% confidence interval (α = 0.05) was performed to evaluate differences in recovery rates between the reported method^25^ and the three newly developed analytical methods. There were no significant differences, as represented in Table 5.

**Table 5:** Results of One–way ANOVA test for the proposed methods and the reported method for the determination of Vonoprazan, Amoxicillin, and Metronidazole in Vonopion^®^ formulation.

	Source	Sum of Squares	df^a^	Mean Square	F	P-value
VON	Between Groups ^**c**^	0.254	3	0.085	0.691	0.583
	Within Groups	0.983	8	0.123		
	Total	1.237	11			
AMX	Between Groups ^**c**^	1.677	3	0.559	0.764	0.546
	Within Groups	5.857	8	0.732		
	Total	7.534	11			
MET	Between Groups ^**c**^	3.246	3	1.082	1.501	0.287
	Within Groups	5.766	8	0.721		
	Total	9.011	11			

^**a**^ Degrees of freedom.

^**b**^ F is the ratio of the mean square to the error mean square.

^**c**^ Between the reported method and the developed methods.

### Greenness and Blueness assessment

The principle of greenness and blueness evaluation has become more significant in developing and validating analytical procedures with the growth of environmental and sustainability concerns. Conventionally developed methods often use more hazardous solvents, generate a lot of waste, and use a lot of energy.

Greenness assessment evaluates analytical methods by the Analytical Greenness measure (AGREE) and the Green Analytical Procedure Index (GAPI) while the blueness assessment evaluates The Blue–Analytical Greenness Index (BAGI), these metrics have been developed to make this assessment easier^45–47^.

### Analytical Greenness metric approach (AGREE)

It is a modern tool designed to evaluate how well an analytical method aligns with the 12 principles of Green Analytical Chemistry. AGREE provides a score from 0 to 1 (where 1 = perfectly green), a Color-coded circular pictogram that visually represents compliance with each principle.

It’s one of the most comprehensive and intuitive tools for greenness assessment of analytical techniques.

### The Green Analytical Procedure Index (GAPI)

It is a semi-quantitative technique that evaluates the greenness of analytical procedures using a color-coded pentagram; pentagram colors represent minimal, moderate, and high environmental impact, respectively, in the green, yellow, and red zones. This thorough method aids in identifying areas that require improvement. It considers factors like sample preparation, reagents, hazards, instrumentation, and waste in evaluating the greenness’s analytical methods.

### The Blue–Analytical Greenness Index (BAGI)

It is a free software tool designed to assess the sustainability and simplicity of analytical methods. It provides two main indicators: a score in which values above 60 indicate high relevance and environmental impact, and an asteroid-shaped pictogram in which darker blue areas represent greater applicability, while white areas indicate lower environmental impact. BAGI offers a clear and practical way to compare analytical methods by combining these visual and numerical evaluations.

As shown in Table 6, the proposed spectrophotometric methods demonstrate more eco-friendly profiles, based on GAPI, AGREE, and BAGI, when compared with the reported reference HPLC methods^25,48^.

**Table 6:** Greenness assessment and evaluation of proposed UV spectrophotometric methods against reported HPLC method.

	GAP1	AGREE-prep	BAGI
Proposed UV spectrophotometric methods			
Reported HPLC method^25^			
Reported HPLC method ^48^			

## Conclusion

Applying UV-spectrophotometric methods offers a robust and efficient approach for the simultaneous analysis of the ternary drug combination comprising Vonoprazan, Amoxicillin, and Metronidazole in pharmaceutical dosage forms and dissolution testing. By offering advanced techniques that resolve the highly overlapping absorption spectra, enabling quantification of individual components. These methods provide accuracy, validation through recovery studies, and statistical comparison with the reference reported method. Beyond its analytical performance, the methods align with green analytical chemistry principles, as they minimize solvent consumption, eliminate hazardous reagents, and reduce energy demands. Underscores its utility in routine analytical laboratories seeking high-throughput, cost-effective alternatives to cinematographic methods. UV-spectrophotometric methods are valuable for pharmaceutical quality control and supporting dissolution profile characterization in pharmacokinetic studies.

## Data Availability

The data that support the findings of this study are available from the corresponding author, upon reasonable request.
